# The prevalence and significance of isolated hepatitis B core antibody (anti-HBc) in endemic population

**DOI:** 10.1186/s13104-019-4287-z

**Published:** 2019-05-06

**Authors:** Chul S. Hyun, Seulgi Lee, William R. Ventura

**Affiliations:** 1 Center for Viral Hepatitis, 35 Van Nostrand Avenue, Englewood, NJ 07631 USA; 2 Holy Name Medical Center, Teaneck, NJ USA; 3 St. Joseph Regional Medical Center, Paterson, NJ USA

**Keywords:** Hepatitis B virus (HBV), Hepatitis B core antibody (anti-HBc), Antiviral prophylaxis, Hepatitis B screening, HBV reactivation

## Abstract

**Objective:**

There are three major serologic markers for hepatitis B virus (HBV) infection: hepatitis B surface antigen (HBsAg); hepatitis B surface antibody (anti-HBs); and hepatitis B core antibody (anti-HBc). HBV screening programs, however, often test only HBsAg and anti-HBs, missing those individuals who have anti-HBc as the only detectable marker. Isolated anti-HBc can represent chronic infection in which HBsAg is not detectable by serology. We, therefore, investigated the prevalence of isolated anti-HBc in an ethnic community at moderate to high risk for HBV infection.

**Results:**

Of 7157 Korean American adults in New Jersey, 2736 (38.2%) lacked anti-HBs, potentially susceptible to HBV. Of these 2736 subjects, 771 subjects had anti-HBc. The prevalence of isolated anti-HBc increased with age: 0.8% (age 21–30); 2.4% (age 31–40); 6.05% (age 41–50); 11.7% (age 51–60); 18.3% (age 61–70); and 24.5% (age 71–91). Similarly, the percentage of the individuals with isolated anti-HBc in anti-HBs lacking subjects showed a striking age dependence. We conclude that serologic HBV screening should include anti-HBc to accurately assess the prevalence of HBV exposure. Serologic screening with only HBsAg and anti-HBs may overestimate the prevalence of non-immune population. It can also underestimate the prevalence of HBV and increase the risk of HBV reactivation during immunosuppression.

## Introduction

HBV infection is a serious global health issue. Approximately 257 million people are currently infected with HBV. The number of people who have had the infection in the past and developed protective antibody, who are convalescent, however, is as high as 2 billion, making hepatitis B one of the most common infections in the world [1, 2].

Although most of the individuals who are in the convalescent phase are protected from the virus, there are many instances when these people can have reactivation under certain circumstances [3, 4]. For instance, individuals with history of past HBV infection, whether they are currently infected or not, may be at risk for reactivation hepatitis during and after immunosuppressive therapy. Reactivation of HBV can potentially lead to fulminant liver failure and/or death [5, 6]. Therefore, it is critical to identify those individuals who may be at risk for reactivation before immunosuppressive therapy.

The US Center for Disease Control and Prevention and other key organizations have recommended that all patients undergoing chemotherapy should be screened for HBV [7–9]. Yet HBV screening for cancer patients is significantly low. A large-scale US study, for instance, has demonstrated that only 16.7% of 10,729 patients who received chemotherapy underwent HBsAg or anti-HBc screening. All these patients were newly diagnosed cancer patients seen at a large tertiary cancer center in the United States. Even among the patients with risk factors, only 19.3% were screened. Furthermore, only 16.9% of Asian patients were screened despite the fact that Asian ethnicity had a highly significant predictor of positive test results [10].

The serologic test of choice for establishing a history of previous infection is hepatitis B core antibody (anti-HBc). Anti-HBc is the earliest antibody to develop in response to HBV infection, appearing as IgM anti-HBc. Anti-HBc typically persists for life as IgG anti-HBc after 6 months of the infection. Thus, IgM anti-HBc is a marker of acute infection while IgG-anti HBc is a marker of past infection. Isolated anti-HBc refers to the presence of IgG anti-HBc in the absence of HBsAg and anti-HBs.

Once the ‘window period’ following acute infection is ruled out, the finding of isolated anti-HBc can signify either remote infection with waning anti-HBs titre without viremia or infection with undetectable level of HBsAg. The latter group of individuals with undetectable HBsAg belongs to occult hepatitis B virus infection (OBI), defined as presence of HBV DNA in the liver of individuals testing HBsAg-negative. OBI has been shown to occur in both absence and presence of anti-HBc and/or anti-HBs. The prevalence of OBI has been noted to be more common in patients at high risk for parenterally transmitted infections such as hepatitis C virus (HCV) and HIV infections [11]. While the isolated anti-HBc may result in a small frequency from a false-positive test, the prevalence of isolated anti-HBc has been as high as 12% in endemic population [12, 13]. Thus, hepatitis B screening without inclusion of anti-HBc can potentially miss a significant number of the individuals with OBI and/or a history of past infection in endemic populations.

Antiviral prophylaxis is recommended for all the individuals with history of past infection to prevent reactivation hepatitis. This includes those individuals with past history of infection with anti-HBs. Preventive antiviral therapy for the patients undergoing chemotherapy could potentially lower the risk for HBV reactivation and the reactivation-associated morbidity and mortality [3, 6].

In a large scale, community-based hepatitis B screening from 7157 Korean Americans, we have demonstrated that 771 (10.8%) had isolated anti-HBc. These 771 individuals represented 28.2% of 2736 subjects, who were not immune, demonstrating history of past HBV infection [14]. The present study further analyzed the prevalence of anti-HBc in various age groups and discussed the significance of these data in prevention of potential HBV reactivation in a high risk population.

## Main text

### Methods

#### Participants

Korean American subjects who lacked anti-HBs were identified from community-based hepatitis B screening events held in the state of New Jersey between December 2009 and June 2015. These screening campaigns were organized by the Center for Viral Hepatitis (CVH) and Holy Name Medical Center. The data on the subjects who were HBsAg and anti-HBs negative were further evaluated for the presence of anti-HBc. Secondly, the prevalence of isolated anti-HBc was investigated in different age groups.

#### Serological screening and survey

This study is a retrospective analysis of 7157 individuals who underwent HBV screening. Serologic screening for hepatitis B included the following tests: HBsAg, anti-HBs, and anti-HBc (IgG). All of the serologic tests employed ADVIA Centaur XPT assay system (Siemens Healthcare Diagnostics, Inc., Tarrytown, NY). Blood samples were collected and processed by phlebotomists, and all the results were reported by physicians. Results of the screenings were provided directly to all the participants.

#### Statistical analysis

Cochran–Armitage trend test was employed to evaluate the significance of linear trend of anti-HBc prevalence with increasing age.

### Results

#### Proportion of isolated anti-HBc in anti-HBs lacking and total populations

All the subjects were Korean American residents currently in New Jersey. 99.7% of the participants were immigrants from Korea. The participants’ ages ranged from 21 to 91, with a mean age of 55. A vast majority (> 85%) of the participants have lived in the United States for a minimum of 10 years.

**Fig. 1:**
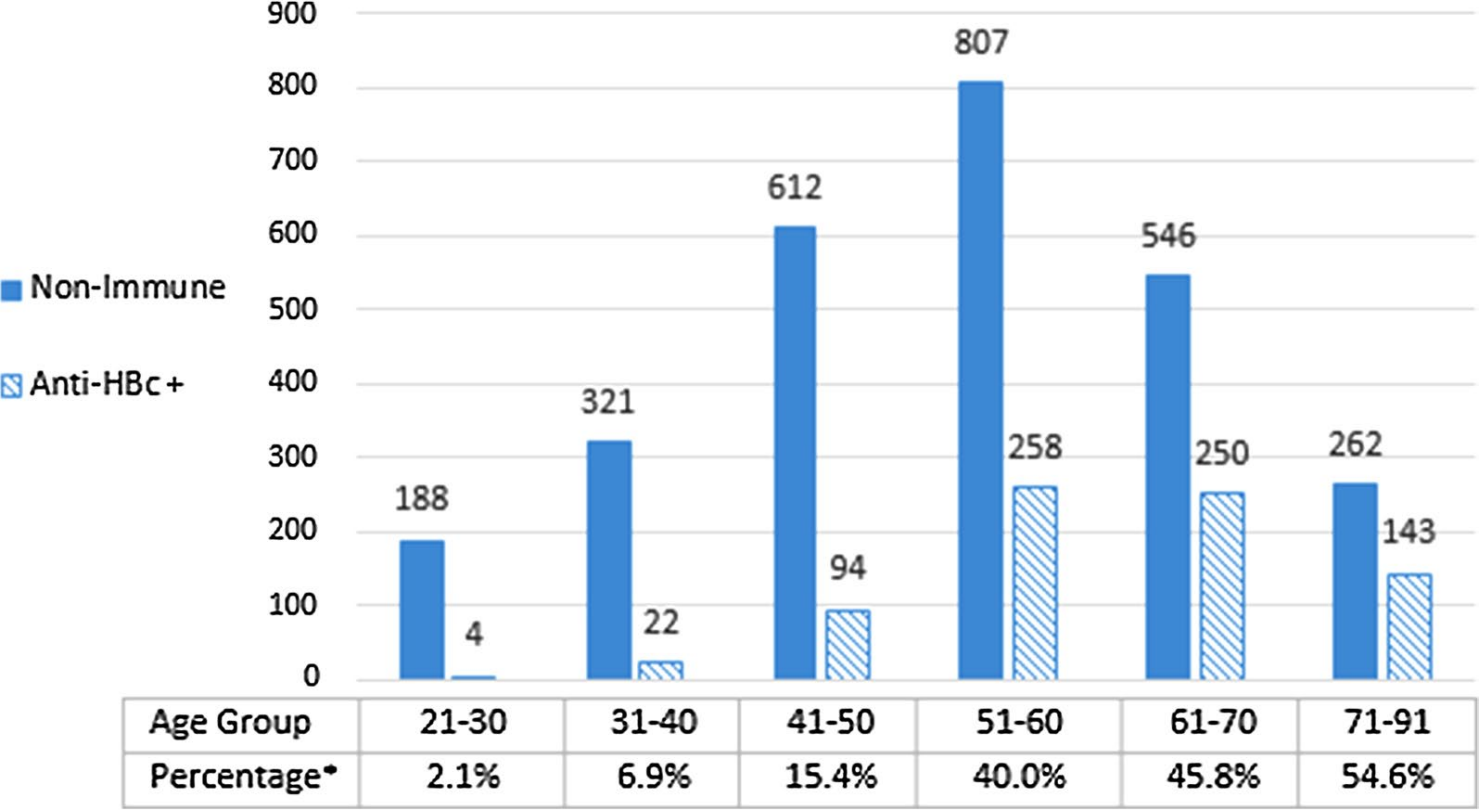
Presence of anti-HBc in different age groups of anti-HBs lacking population. Asterisk: Percentage refers to a proportion of those with isolated anti-HBc in the subjects lacking anti-HBs in the indicated age group

Of 7157 Korean American adults initially tested in community-based screening events, 2736 (38.2%) were found susceptible to HBV, lacking protective anti-HBs [14]. Of these 2736 subjects lacking anti-HBs, 771 subjects had anti-HBc (Fig. 1). Thus, a total of 771 subjects (10.8%) out of 7157 had isolated anti-HBc. Of 771 subjects with isolated anti-HBc, 385 were males, 370 females, and 16 unknown. The rate of isolated anti-HBc was significantly higher in males (13.00%) as compared to females (8.94%) (p < 0.01). The evaluation on the proportion of isolated anti-HBc among those lacking anti-HBs showed a significant age dependent increase in the percentage of isolated anti-HBc: 2.1% (age 21–30); 6.9% (age 31–40); 15.4% (age 41–50); 40% (age 51–60); 45.8% (age 61–70); and 54.6% (age 71–91) (Fig. 1).

#### Prevalence of anti-HBc in different age groups

We investigated the prevalence of isolated anti-HBc in the total population of subjects, and the results demonstrated an impressive age-dependent increase in isolated anti-HBc (Table 1). There was a significant linear trend of increasing anti-HBc prevalence with increasing age (Cochran–Armitage trend test z = 18.4, two sided p < 0.0001).

**Table 1:** Prevalence of anti-HBc in different age groups

Age groups: % (case/total)						
21–30	31–40	41–50	51–60	61–70	71–91	Total
8% (4/508)	2.4% (22/910)	6.0% (94/1559)	11.9% (258/2175)	18.4% (250/1359)	24.5% (143/584)	10.9% (771/7059)

### Discussion

Our previous study involving a community-based hepatitis B screening from 7157 Korean Americans (age 21–91) in New Jersey between December 2009 and June 2015 showed that 171 (2.4%) were HBV infected, 4250 (59.4%) were immune, and 2736 were susceptible to HBV [14]. Of 4250 immune subjects, 2319 were recovered from past infection, and 1931 were vaccinated.

The current study demonstrated that 771 of 2736 subjects, who were not immune, had anti-HBc, demonstrating past HBV infection. The large proportion of isolated anti-HBc among the anti-HBs lacking population (Fig. 1) demonstrates the need for including anti-HBc in every hepatitis B screening tests.

Given the low rate of false positive anti-HBc, there are several potential explanations for isolated anti-HBc. First, a small number of the subjects with isolated anti-HBc might have had acute infection when the so-called ‘window period’ between HBsAg disappearance and appearance of anti-HBs took place. Second, isolated anti-HBc may represent a remote resolved infection with an undetectable level of anti-HBs. Third, some patients with isolated anti-HBc may have OBI with undetectable level of HBsAg [11, 12, 15–17]. These OBI patients with isolated anti-HBc, albeit small, may have detectable HBV DNA and may be at risk for liver cirrhosis and hepatocellular carcinoma. The current study, however, has not determined HBV DNA levels in isolated anti-HBc participants and, therefore, cannot directly comment on the OBI. With a low rate of acute hepatitis B infections in the United States [18], the increasingly high prevalence seen in the older subjects in this study is congruent with the notion that most of these subjects had anti-HBs in the past which waned in time. In fact, a majority of patients with isolated anti-HBc have been shown to respond to HB vaccine with anti-HBs formation [13, 15].

Many of the hepatitis B screening programs employed in community screening campaigns and private practice settings test only HBsAg and anti-HBs, omitting anti-HBc. These screenings may fail to reveal a large group of individuals with isolated anti-HBc, who have had an exposure to HBV in their past. Thus, anti-HBc test should be included in every routine Hepatitis B screening. The proportion of subjects who had isolated anti-HBc in the absence of anti-HBs represents previously infected individuals and demonstrates an impressive increment with age (Fig. 1). According to our data, nearly half of the anti-HBs lacking Korean American population above age 60 may be mistaken for not having past infection if they were tested only for HBsAg and anti-HBs. With the number of potential HBV reactivations and the significant rise in hepatocellular carcinoma in the United States, the possible underestimation of HBV exposure and infection in immigrant populations at risk would be an important public health concern.

All the patients who may appear to have recovered from past infection (HBsAg-negative, anti-HBs negative or positive, and anti-HBc positive) may still be at risk for HBV reactivation on immunosuppressive therapy [3–5, 11]. Although HBV DNA may not be detectable in serum, HBV may persist in the liver [11, 16]. As HBV replication is controlled by the immune system, a significant degree of immunosuppression can reactivate dormant HBV and cause serious outcome. HBV reactivation in the patients with isolated anti-HBc can be diagnosed upon the appearance of HBsAg and/or HBV DNA. Potentially fatal HBV reactivation can be prevented by prophylactic antiviral therapy. It is recommended to initially stratify the risk for HBV reactivation on each patient with the extent and types of immunosuppressive therapy before deciding on antiviral prophylaxis [3, 10].

Failure to identify isolated anti-HBc in cancer patients could potentially bring devastating outcomes. In consideration of the striking link between advanced age and incidence of cancer, the omission of anti-HBc in HBV screening in endemic population can cause a greater impact in older populations. Our observation that the prevalence of isolated anti-HBc is significantly higher in older populations emphasizes the need of screening anti-HBc in the same population.

## Limitations

There are several limitations in our study. First, the participants may not necessarily represent the overall Korean American population in the United States. While the participants consisted of individuals from mixed demographic backgrounds, the sample may not be a true random representative of Korean American population. Future studies employing subjects with a wider spectrum of ages and socio-economic classes would help determine the accurate status of HBV in this ethnic group [19, 20]. Second, many of the subjects possibly underwent the screening knowing their HBV status, possibly skewing the result to one side. For instance, if a greater number of subjects with history of past infection relative to other subjects underwent the screening, the isolated anti-HBc prevalence could be overestimated. Third, acute hepatitis B infection has not been ruled out in the subjects with isolated anti-HBc. However, with a declining incidence of acute hepatitis B infection in the United States, the proportion of isolated anti-HBc in the window period would be expected to be minimal. Fourth, HBV DNA tests were not performed on the isolated anti-HBc positive participants. Thus, the proportion of OBI in these participants could not be determined. Last, HCV and HIV co-infections could have potentially increased the prevalence of isolated anti-HBc. However, Asian American rates of HIV and HCV infections are known to be very low [21].

## Data Availability

The datasets used and/or analysed during the current study are available from the corresponding author on reasonable request.

## Abbreviations

HBV: hepatitis B virus
CHB: chronic hepatitis B
HBsAg: hepatitis B surface antigen
anti-HBs: hepatitis B surface antibody
anti-HBc: hepatitis B core IgG antibody
HCV: hepatitis C virus
HIV: human immunodeficiency virus
